# Effects of Obstructive Sleep Apnea on Cardiac Function and Clinical Outcomes in Chinese Patients with ST-Elevation Myocardial Infarction

**DOI:** 10.1155/2014/908582

**Published:** 2014-02-17

**Authors:** Baoxin Liu, Rong Guo, Shunping Zhou, Shuanshuan Xie, Ke Wang, Yawei Xu

**Affiliations:** ^1^Department of Cardiology, Shanghai Tenth People's Hospital, Tongji University School of Medicine, 301 Middle Yanchang Road, Shanghai 200072, China; ^2^Department of Respiratory Medicine, Shanghai Tenth People's Hospital, Tongji University School of Medicine, 301 Middle Yanchang Road, Shanghai 200072, China

## Abstract

*Aim*. The objective of this study was to investigate the influence of OSA on cardiac function in Chinese patients with ST-elevation myocardial infarction (STEMI) and determine the prognostic impact of OSA among these patients. *Methods*. In this retrospective study, 198 STEMI patients were enrolled. Doppler echocardiography was performed to detect the effect of OSA on cardiac function. Major adverse cardiac events (MACE) and cardiac mortality were analyzed to determine whether OSA was a clinical prognostic factor; its prognostic impact was then assessed adjusting for other covariates. *Results*. The echocardiographic results showed that the myocardium of STEMI patients with OSA appeared to be more hypertrophic and with a poorer cardiac function compared with non-OSA STEMI patients. A Kaplan-Meier survival analysis revealed significantly higher cumulative incidence of MACE and cardiac mortality in the OSA group compared with that in the non-OSA group during a mean follow-up of 24 months. Multivariate Cox regression analysis revealed that OSA was an independent risk factor for MACE and cardiac mortality. *Conclusion*. These results indicate that the OSA is a powerful predictor of decreased survival and exerts negative prognostic impact on cardiac function in STEMI patients.

## 1. Introduction

Obstructive sleep apnea (OSA) is the most common clinical type of sleep-related breathing disorders, which is characterized by recurrent episodes of complete or partial obstruction of the upper airway, leading to increased negative intrathoracic pressure, habitual snoring, sleep fragmentation, excessive daytime sleepiness, and intermittent hypoxia during sleep [1]. Most OSA patients may also complain of excessive daytime sleepiness or insomnia, nocturia, and morning headaches. Research on the current epidemiology of OSA demonstrated that one-third of sleep studies showed some degree of OSA. Among middle-aged adults, approximately 13% of men and 6% of women have moderate to severe OSA [2, 3]. Asia is the most heavily populated continent, with some groups living in an underdeveloped environment. OSA prevalence in Asia ranged from 3.7% in the Japanese study [4] to 88.81% in the Chinese study [5]. This significant difference may be attributed to the different methods and populations studied with a greater body mass index (BMI) and older age in the latter study, since older age, greater BMI, and diabetes mellitus were associated with OSA. Overall, it is estimated that 50% to 60% of obese patients with metabolic syndrome have OSA [6, 7]. The proportion is even higher in obese patients with diabetes mellitus [8].

OSA has recently been recognized as a risk factor in the cause and promotion of cardiovascular and cerebrovascular diseases, including coronary artery disease (CAD), hypertension, heart failure, arrhythmias, and stroke [9–11]. Most importantly, OSA has been reported to be an important factor in the occurrence of acute myocardial infarction (AMI) [12]. ST-elevation myocardial infarction (STEMI) is the most serious and common clinical type of AMI which is characterized by ST-segment elevation in relevant leads on electrocardiogram (ECG). The pathogenesis of STEMI is thrombosis and occlusion of coronary arteries, which results from the formation and rupture of vulnerable coronary atherosclerotic plaques. Without prompt and complete restoration of flow in the infarct artery, STEMI could lead to serious consequences. In China, STEMI has now been a major public health problem for its high morbidity and mortality following the formation of the aging society in recent years [13].

The underlying pathophysiological mechanism between OSA and STEMI was complex. Severe nocturnal hypoxemia caused by OSA, long-term hypoxia-induced injury in endothelial, hemodynamic changes, and neurohormonal abnormality will increase the risk of formation and rupture of atherosclerotic plaques, which may finally result in STEMI. Moreover, the decrease in oxygen desaturation in OSA patients was closely related to ST-segment depression, angina and AMI, even if they were not clinically defined as CAD patients [14, 15]. Previously studies have reported that 65.7% of patients presenting with STEMI had undiagnosed OSA and OSA was also an independent risk factor for impaired recovery of left ventricular function after myocardial infarction [16, 17]. However, the effect of OSA on cardiac function and clinical outcomes has not been well studied. Existing reports on the role of OSA as an adverse prognostic marker after STEMI were also conflicting [18, 19]. The objective of this study is to investigate the relationship between OSA and cardiac function in Chinese patients with STEMI and determine the effect of OSA on the prognosis of STEMI.

## 2. Methods and Materials

### 2.1. Study Design and Patient Population

From February 2012 to April 2013, a total of 198 patients diagnosed as STEMI in our department were enrolled in this retrospective study. Standard 12-lead ECGs, data on cardiac biomarker, echocardiographic, and coronary angiography (CAG) findings were collected. The demographics, including history of significant CAD (prior myocardial infarction or typical angina pectoris), previous percutaneous coronary intervention (PCI), or coronary artery bypass grafting (CABG), and risk factors for CAD (age, gender, BMI, hypertension, tobacco use, drinking habit, diabetes mellitus, chronic kidney disease, and lipid disorders) were documented. Hypertension was defined as systolic blood pressure/diastolic blood pressure ≥140/90 mmHg in the supine position, or the use of antihypertensive medication. Diabetes mellitus was identified by a fasting plasma glucose ≥ 7.0 mmol/L, or random plasma glucose ≥ 11.1 mmol/L, or if patients received therapy using insulin or oral medications for diabetes. Chronic kidney disease (CKD) was defined as an eGFR < 60 mL/min/1.73 m^2^. Lipid disorders were defined as the presence of total cholesterol ≥ 5.7 mmol/L, LDL ≥ 3.6 mmol/L, HDL < 1.04 mmol/L, or current lipid-lowering medication use. Tobacco use and drinking habit, either at time of the interview or in the past, were recorded. Medications including antiplatelet drugs, statins, *β*-blockers, angiotesin converting enzyme inhibitors (ACEI), angiotensin II receptor blockers (ARB), calcium channel blockers (CCB), diuretics, and digitalis were also noted. According to the prescribed medication recorded in the study, the dual antiplatelet therapy (DAPT) adherence was evaluated. DAPT adherence referred to the use of scheduled clopidogrel plus aspirin medication for at least 1 year after discharge.

Patients who had a confirmed diagnosis of STEMI were eligible to participate in this study if (1) the age ranges from 18 to 80 years; (2) patients were able to understand the study content and provide consent; (3) patients were willing to accept the necessary follow-up, therapy, and laboratory examination. All patients were diagnosed based on symptoms, positive tests for biomarkers of necrosis, ECG, echocardiographic, and CAG results. The diagnosis of STEMI was according to the World Health Organization definition of myocardial infarction (2008-09 revision) [20]. The exclusion criteria included (1) patients aged over 80 years, (2) patients with non-ST elevation myocardial infarction; (3) patients with a life expectancy of less than 12 months; (4) pregnant and lactating women, (5) patients who were unable to understand the study content, or provide consent, (6) patients with already diagnosed OSA before STEMI.

The study was approved by the institutional ethics committee of Shanghai Tenth People's Hospital, and all enrolled patients gave informed written consent to the study.

### 2.2. Overnight Sleep Measurement

An overnight polysomnography (PSG) was performed using a digital system (Embla S4500, Embla Systems, Broomfield, CO, USA) at the sleep monitoring room during the patient's habitual sleep time. The following physiological parameters were measured simultaneously and continuously: electroencephalogram, electrooculogram, and chin electromyogram were recorded to evaluate the stage of sleep. Sleep states and arousal were scored on the basis of standard criteria. Nasooral air flow, thoracoabdominal respiratory movement, snoring, and body position were used to determine the type of apnea and respiratory mechanics instability (RMI). Oxyhemoglobin saturation (SpO_2_, percentage of available hemoglobin that is saturated with oxygen) was estimated by pulse oximetry and electrocardiograph recordings were taken from a single lead.

All PSG results were analyzed by 2 trained technicians with no knowledge of the clinical characteristics of the patients. According to the American Academy of Sleep Medicine criteria [21], an apnea was defined as >90% decrease in the airflow signal lasting for at least 10 seconds and a hypopnea as ≥30% decrease in airflow signal, with a relevant reduction in SpO_2_ (≥4% of baseline at the nadir) lasting for more than 10 seconds. Desaturation time was calculated through the cumulative percentage of sleep time with SpO_2_ ≤ 90%. Oxygen desaturation index was calculated by dividing the total number of oxygen desaturations by the total sleep time, independent of airflow or thoracoabdominal movement. Obstructive apnea was defined as the absence of air flow despite respiratory movement or effort. Central sleep apnea was defined as the absence of both air flow and respiratory movement. The apnea-hypopnea index (AHI) was defined as the average number of apneas and hypopneas per hour of sleep, and an AHI ≥ 5 established the diagnosis of OSA regardless of associated symptoms.

### 2.3. Doppler Echocardiography

Investigations of cardiac structure and function were performed at baseline (on admission) after discharge at 1, 6, 12, and 24 months. Each parameter was the mean value averaged from four measurements. Echocardiographic examinations were performed at rest, with the patient semirecumbent in the left lateral position. All scans were performed by 2 experienced sonographers, using a GE Vivid 7 (GE Healthcare, Piscataway, NJ, USA) ultrasound machine with a M4S (1.7–3.4 MHz) transducer, and reported by cardiologists with advanced training in echocardiography. Left ventricular measurements were analysed using the M-mode from the parasternal long axis according to the American Society of Echocardiography guidelines [22]. Left ventricular mass (LVM) and ejection fraction (LVEF) were also calculated from M-mode measurements [23, 24].

The pulsed Doppler sampling volume was placed between the tips of the mitral valve leaflets to obtain maximum filling velocities in passive end expiration by using a 3–5 mm sample volume. A standardized loop of 10 cardiac cycles was downloaded to the computer for analysis of the peak of early diastolic velocities (peak E), the peak of late diastolic velocities (peak A), the deceleration time of the peak E velocity (DT), and isovolumic relaxation time (IVRT). Pulsed wave Doppler tissue imaging (DTI) was acquired in the apical 4-chamber view placed over the myocardium, on the septum, at the level of the mitral annulus. Systolic motion (s′ wave) and early (e′) and late diastolic (a′) mitral annulus velocities were obtained. The e′ wave velocities from the septal and lateral walls were averaged and the ratio of the transmitral E wave to the average e′ velocity (E/e′ ratio) was calculated as an indicator of left ventricular filling pressure. All measurements were made offline on 3 separate beats and then averaged for all parameters.

### 2.4. CAG

All enrolled patients were confirmed by CAG. Of these patients, 94 underwent angiography during the first 24 hours of the STEMI episode, and 105 underwent CAG within 2 to 14 days after the acute episode. The location of coronary lesions, the number of stenosed arteries, and the degree of stenosis were recorded for each patient.

All images of the coronary tree were obtained in routine standardized projections with an Axiom Artis system (Siemens AG, Henkestrasse, Erlangen, Germany). Multiple views of each coronary artery were obtained and all images of the coronary tree were recorded as appropriate and reproduced at the time of follow-up angiography. Coronary artery stenoses were quantitatively assessed with the ST-ADS software package (Siemens AG, Henkestrasse, Erlangen, Germany) after direct intracoronary injection of 2.5–5 mg nitroglycerin into the left and right coronary arteries to eliminate coronary spasm. The initial and follow-up coronary angiograms were obtained in the same projection, and the images were quantitatively assessed by a single cardiologist.

Adachi/Bianchi classification was used to categorize the particular vasculature types: classic coronary artery, neither artery is dominating; dominant right coronary artery; dominant left coronary artery [25]. The severity of stenosis was graded by using the Coronary Angiogram Analyzing System II (CAAS II; Pie Medical, Maastricht, The Netherlands) [26]. Preprocedural and postprocedural coronary flow over the culprit lesion was graded according to the Thrombolysis in Myocardial Infarction Trial (TIMI) criteria, and collateral circulation was classified according to the criteria proposed by Morrow et al. [27–29]. Multivessel CAD was defined as the presence of lesions in ≥3 coronary vessels. Single-vessel disease referred to occlusion in the main and secondary branch of a vessel [30].

### 2.5. Follow-Up and Endpoints

All patients were followed up at 1, 3, 6, 12, 18, and 24 months after discharge. The medical history was taken, and prescribed medication was evaluated by medical review, and relevant examinations were performed if necessary. Patients were also asked carefully at each follow-up regarding the presence or absence of relevant symptoms. The length of follow-up was measured from the initial cardiac event.

Clinical follow-up data were gathered by reviewing outpatient records. Study endpoint is the 2-year major adverse cardiac events (MACE). The MACE consisted of a composite of (1) cardiac death; (2) a recurrent nonfatal myocardial infarction; (3) clinically driven target lesion revascularization (TLR) or target vessel revascularization (TVR). Cardiac death was defined as any death caused by a primary cardiovascular problem, including sudden and nonsudden cardiac death [31]. Clinically driven TLR was considered to be ischemia-driven if the target lesion diameter stenosis was ≥50% by quantitative analysis with either electrocardiographic changes at rest or a positive functional study in the distribution of the target lesion, or ≥70% with recurrent symptoms only. The target lesion was defined as the coronary segment containing the stent plus a 5 mm proximal or distal margin [32]. TVR was defined as the need for either PCI or CABG of the initial vessel intervened upon and excludes subsequent revascularization of a newly diseased or previously diseased vessel(s) [33].

### 2.6. Statistical Analyses

Results were expressed as mean ± standard deviation (SD) for continuous variables and frequencies for categorical variables. Differences between groups were examined by nonparametric test and chi-square test for continuous and categorical variables, respectively. The effect of OSA on clinical outcome was assessed with the use of a multivariate Cox proportional hazards model. Other variables that were significantly associated with outcomes were entered into the model in a stepwise procedure. Cumulative incidence of MACE and cardiac mortality curves were constructed using the Kaplan-Meier method and compared using the log-rank test. An alpha value of 0.05, corresponding to a *P* value < 0.05, served as criterion for establishing statistical significance. The 95% confidence intervals of the hazard ratio were reported for all of the significant risk factors. Analysis was performed using SPSS for Windows (SPSS Inc., Version 16.0, Chicago, IL, USA).

## 3. Results

### 3.1. Demographics and Clinical Data

**Figure 1:**
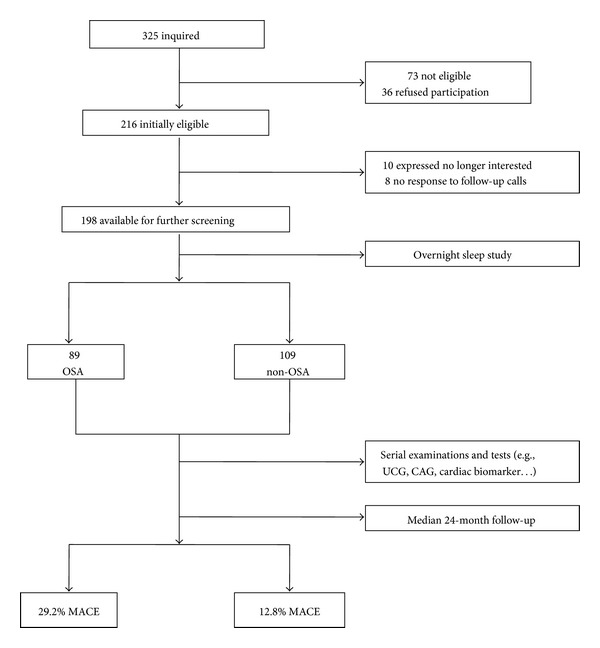
Patient flow and main outcomes. OSA, obstructive sleep apnea; UCG: ultrasonic cardiogram; CAG: coronary angiography; MACE: major adverse cardiac events.

**Figure 2:**
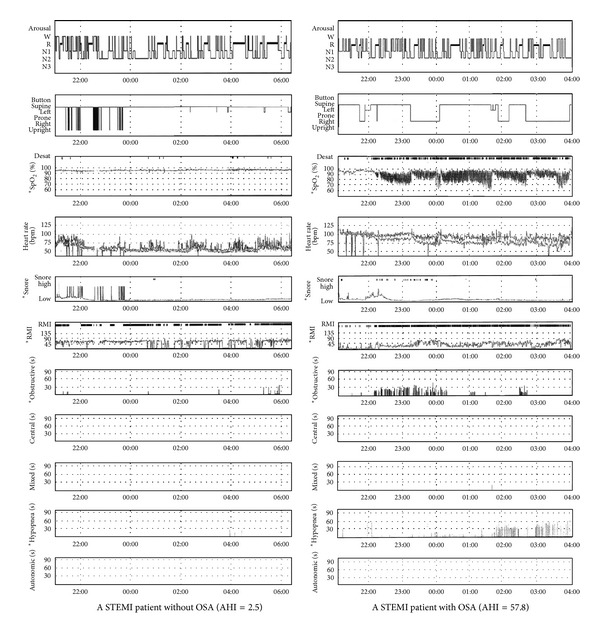
Representative overnight sleep study results in the OSA and non-OSA groups. SpO_2_, oxyhemoglobin saturation; RMI, respiratory mechanics instability; STEMI, ST-elevation myocardial infarction; AHI, apnea-hypopnea index. *refers to several OSA-related indexes which showed significant difference between two patients.

Among the 325 STEMI patients inquired initially, 73 were not eligible and 36 refused to participate in the study. Over a period of follow-up, 10 patients were no longer interested and 8 patients were without response to follow-up calls. According to the different PSG results of enrolled 198 patients, 89 were classified into OSA group and 109 into non-OSA group (Figure 1). The PSG result of one of the OSA group and the non-OSA group has been shown in Figure 2. The mean age of finally enrolled 198 patients is 61.5 ± 7.3. The demographic comparison and drug therapy between two groups were summarized in Table 1. Most of CAD risk factors such as age, gender, hypertension, diabetes mellitus, lipid disorders, CKD, and tobacco use were not significantly different between two groups. However, the incidence of prior CABG was significantly higher in the OSA group, compared with the non-OSA group (*P* = 0.039). The mean abdominal circumference and BMI of OSA group were both significantly higher than those of non-OSA group (*P* = 0.046, *P* < 0.001, resp.). Moreover, socioeconomic status analysis demonstrated that 100 patients of OSA group were in medium to high subgroups, and 71 patients of non-OSA group were in medium to high subgroups (*P* = 0.048).

### 3.2. CAG Results

**Figure 3:**
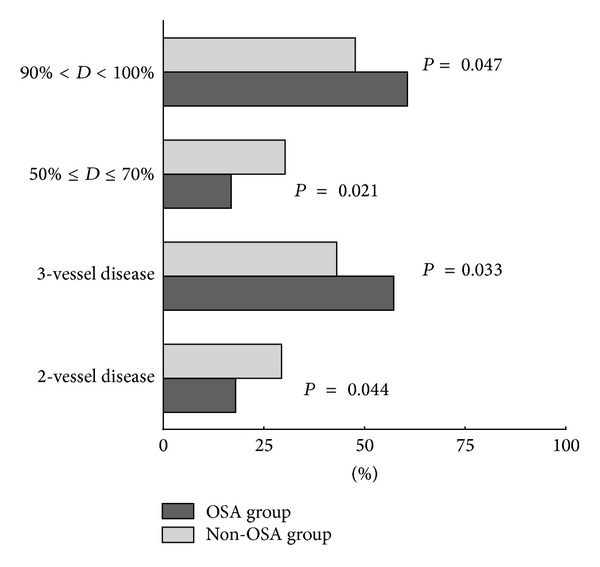
The comparison of coronary lesions and coronary stenosis between the OSA and non-OSA groups reached statistical significance. OSA: obstructive sleep apnea.

The CAG results of enrolled 198 patients were outlined in Table 2. Overall, 10 patients showed dominant left coronary artery, 167 showed dominant right coronary artery, and 21 showed classic coronary artery. 51 patients were with single-vessel disease and 16 with double vessels disease. The proportion of patients with double vessels disease was higher in non-OSA group (*P* = 0.044; Figure 3). The incidence of 3-vessel disease was higher in the OSA group (*P* = 0.001; Figure 3). Similarly, severe and mild degrees of coronary stenosis showed significant difference between two groups. The proportion of patients with stenosis of 50%–70% was higher in non-OSA group (*P* = 0.021; Figure 3). The incidence of coronary stenosis of ≥90% was quite higher in OSA group (*P* = 0.047; Figure 3).

### 3.3. OSA Is Possibly Associated with Cardiac Function

**Figure 4:**
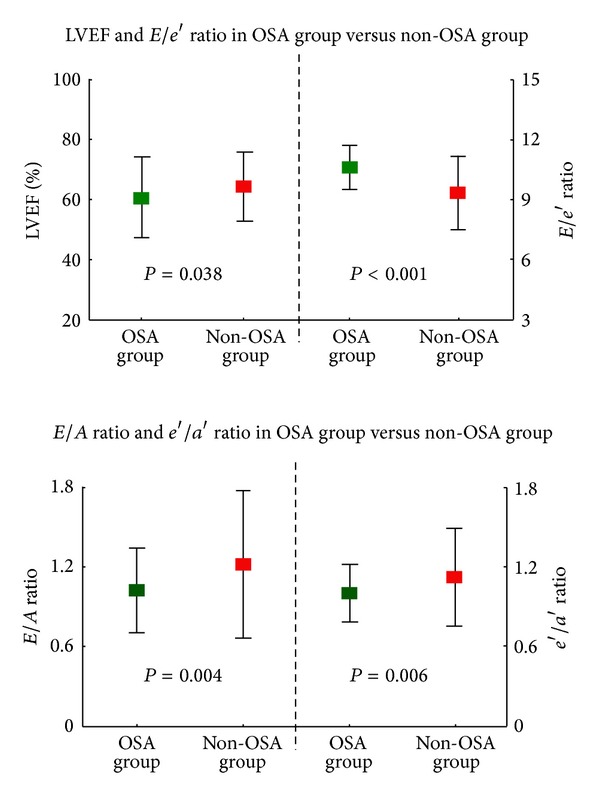
The comparison of several representative indexes of cardiac function between the OSA and non-OSA groups. LVEF: left ventricular ejection fraction; OSA: obstructive sleep apnea.

The cardiac structure and function of the study subjects were shown in Table 3. According to the echocardiographic parameters, whether these were conventionally or DTI-derived, no significant differences were seen to exist between patients with and without OSA in left atrial dimension (LAD), left ventricular end-diastolic dimension (LVDd), left ventricular end-systolic dimension (LVDs), wall motion abnormality (WMA), peak E, and s′. However, LVM, IVS (thickness of interventricular septum), and PWT (thickness of posterior wall) were significant higher in OSA group, compared with non-OSA group (*P* = 0.012, *P* = 0.015, and *P* = 0.045, resp.). Most indexes of cardiac function showed statistic significance between two groups. The mean LVEF in non-OSA group (64.4 ± 11.4%) was significantly higher than that of non-OSA group (*P* = 0.038; Figure 4), which showed a better systolic function in non-OSA group. Similarly, the diastolic function of two groups also showed significant difference. E/e′ ratio, which is a preferable index of diastolic function compared with E/A ratio [34], was better in non-OSA group (*P* < 0.001; Figure 4). Other indexes such as DT, e′, a′, and e′/a′ ratio also showed a better cardiac function in non-OSA patients.

### 3.4. OSA as an Important Factor in Clinical Outcomes

**Figure 5:**
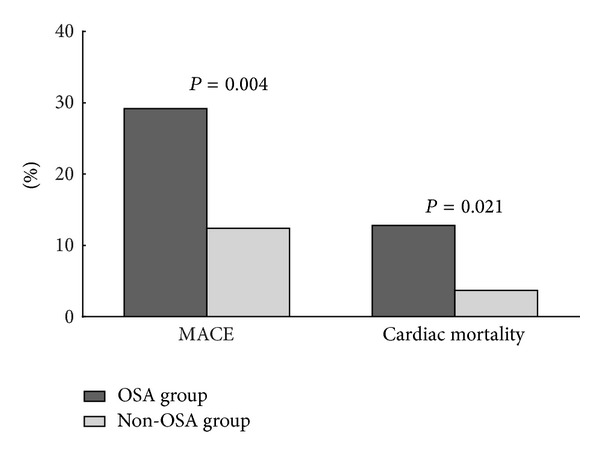
Cardiac mortality and incidence of MACE in patients with and without OSA. MACE: major adverse cardiac events; OSA: obstructive sleep apnea.

**Figure 6:**
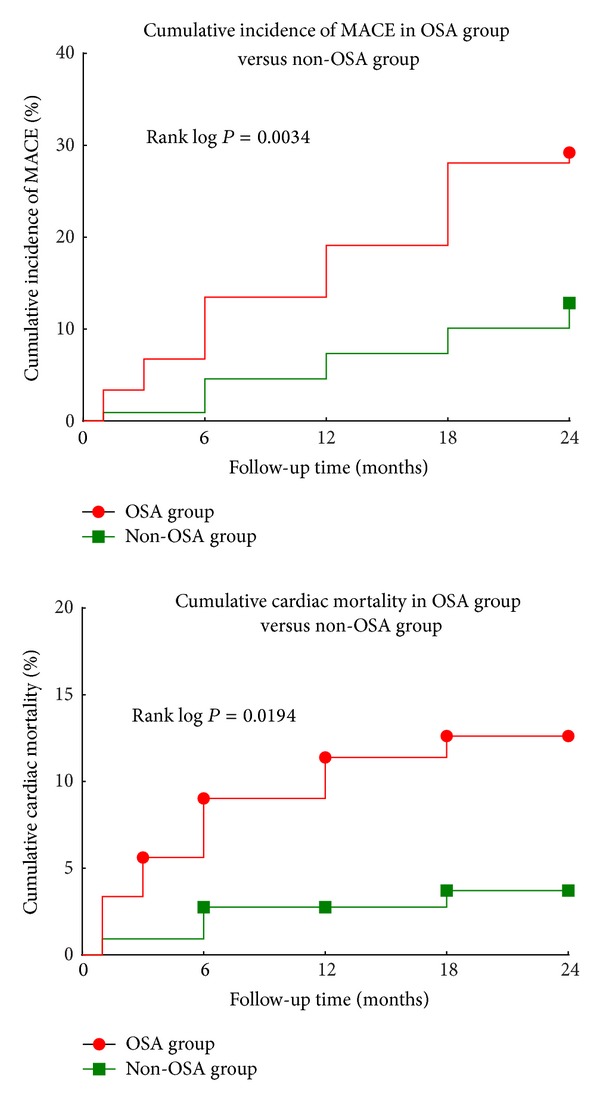
Kaplan-Meier analysis showing cumulative cardiac mortality and incidence of MACE in the OSA and non-OSA groups. MACE: major adverse cardiac events; OSA: obstructive sleep apnea.

**Figure 7:**
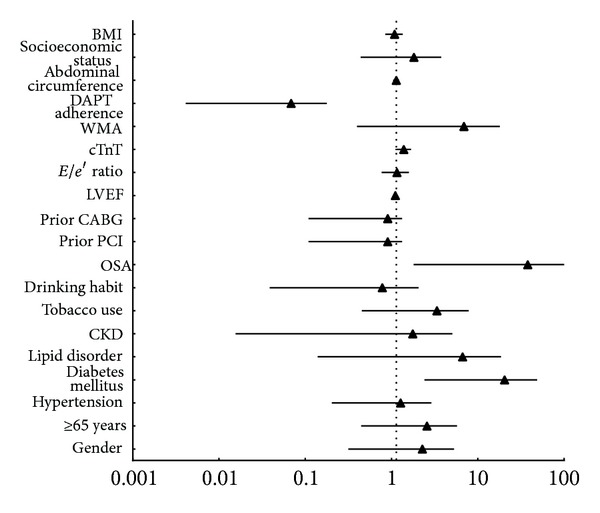
Primary outcome of cardiac mortality. CKD: chronic kidney disease; OSA: obstructive sleep apnea; PCI: percutaneous coronary intervention; CABG: coronary artery bypass grafting; LVEF: left ventricular ejection fraction; cTnT: cardiac troponin T; WMA: wall motion abnormality; DAPT: dual anti-platelet therapy; BMI: body mass index.

**Figure 8:**
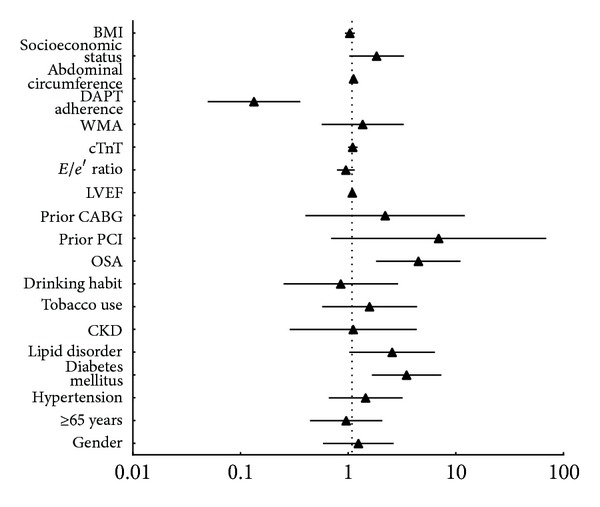
Primary outcome of MACE. CKD: chronic kidney disease; OSA: obstructive sleep apnea; PCI: percutaneous coronary intervention; CABG: coronary artery bypass grafting; LVEF: left ventricular ejection fraction; cTnT: cardiac troponin T; WMA: wall motion abnormality; DAPT: dual antiplatelet therapy; BMI: body mass index.

The average time of follow-up was 21.5 ± 6.0 months. Cardiac mortality (*n*; %) was higher in the OSA group (11; 12.4%) compared with the non-OSA group (4; 3.7%). The proportion of patients with a cardiac event (*n*; %) was also higher in the OSA group (26; 29.2%) compared with the non-OSA group (14; 12.8%; Figure 5). A Kaplan-Meier survival analysis revealed significantly higher cardiac mortality for the OSA group (mean 21.8 months, 95% CI 22.8–24.0) compared with the non-OSA group (*P* = 0.019, Figure 6). Similarly, the Kaplan-Meier survival analysis for MACE showed significantly higher cumulative incidence in the OSA group (mean 20.1 months, 95% CI 18.6–21.6) as compared with the non-OSA group (*P* = 0.003, Figure 6). The multivariate Cox regression analysis showed a significant relationship between OSA and cardiac mortality after adjusting for other risk factors: old age, gender, hypertension, BMI, cTnT, lipid disorders, diabetes mellitus, tobacco use, drinking habit, prior PCI, prior CABG, LVEF, E/e′ ratio, WMA, DAPT adherence, and socioeconomic status (Table 4). The OSA group had higher risk of cardiac death than the non-OSA group (Figure 7). A similar analysis for MACE showed that OSA was an independent risk factor after adjusting for other risk factors, including old age, gender, hypertension, BMI, cTnT, lipid disordoers, diabetes mellitus, tobacco use, drinking habit, prior PCI, prior CABG, LVEF, E/e′ ratio, WMA, DAPT adherence, and socioeconomic status (Table 4). The non-OSA group had a lower risk of major cardiac events than the OSA group (Figure 8). The diabetes mellitus, OSA, and DAPT adherence were all considered risk factors for patients who had significantly lower event-free survival for MACE and cardiac mortality. A patient with OSA and abnormal blood glucose was more likely to have cardiac events than those without OSA and diabetes mellitus.

## 4. Discussion

In this retrospective study to evaluate the effect of OSA on cardiac function and clinical outcomes in STEMI patients, OSA showed a relatively high prevalence in STEMI patients with a proportion of 44.9%. This data is in accordance with the proportion reported by previous studies [35, 36]. Our study showed that a greater BMI and abdominal circumference were associated with OSA (*P* < 0.001, *P* = 0.046), since the BMI and abdominal obesity were reported as important factors in occurrence of OSA [37–39]. Furthermore, the socioeconomic status was correlated with OSA in our study (*P* = 0.048). This may be attributed to the unhealthy dietary habits which may result in abdominal obesity following the elevation of the socioeconomic status in nowadays China.

According to our echocardiographic results, the myocardium of STEMI patients with OSA appeared to be more hypertrophic than that of non-OSA STEMI patients (Table 3); thus the cardiac function could be influenced. Although most indexes of cardiac function were in the normal range, the cardiac function still seemed to be relatively poorer in OSA group as compared with non-OSA group. Apart from the influence of OSA on systolic function reported as aforementioned [17], the diastolic function was also hugely affected by OSA in patients with STEMI. This may also affect the clinical outcomes in the long run. Moreover, approximately 12.4% of patients with OSA but only 3.7% patients without OSA died during a median follow-up of 24 months. Even if adjusting for other cardiac risk factors, diabetes mellitus, DAPT adherence, and OSA were statistically associated with the recurrence of acute myocardial infarction, cardiac death, or revascularization in patients with STEMI. Therefore, our study confirmed that OSA is an independent predictor of cardiac mortality (*P* = 0.019) and major adverse cardiac events (*P* = 0.003) in patients presenting with STEMI, during a mean follow-up of 24 months.

OSA as a risk factor in occurrence of STEMI has been established. However, the exact relationship between OSA and cardiac function in Chinese patients with STEMI and the effect of OSA on the prognosis of STEMI are still being investigated. Previous studies on the prognostic impact of OSA on acute coronary syndrome were limited and conflicting. Peled et al. [15] found that OSA was an independent predictor of adverse cardiac events and nocturnal ischemic events in patients with ischemic heart disease. Hanly et al. [14] reported that OSA was related with recurrence of angina and AMI in STEMI patients. However, these studies were conducted more than 10 years ago, and medical therapy for coronary artery disease has refined tremendously during this period. Mehra et al. [18] reported that OSA was associated with high prevalence of clinically driven TVR rate in 8 months after PCI for acute coronary syndrome. In contrast, Yumino et al. [19] argued that, despite a high prevalence detected, OSA exerted no significant influence on clinical outcomes in STEMI patients. Moreover, these studies failed to provide us relevant evidence on the impact of OSA on cardiac function in the patients with STEMI. Studies on cardiac function of STMEI patients with OSA were also limited. Nakashima et al. [17] have pointed out that OSA could inhibit the recovery of left ventricular function in AMI patients who underwent PCI. Several studies also demonstrated that OSA impaired left atrial wall compliance and passive contraction and thus placed a functional burden to left atrial, which could finally result in arterial hypertension and left atrial remodeling [40, 41]. However, these studies are limited by small sample size, brief follow-up, and inclusion of heterogeneous population. Our study firstly showed report on the association of OSA with cardiac function and long-term clinical outcomes in Chinese STEMI patients, which may also have potential diagnostic and therapeutic implications. The overnight sleep study may be helpful in identifying high risk STEMI patients who are likely to have worse long-term prognosis, and properly treatment of OSA could also have beneficial effects on long-term cardiovascular outcomes.

The mechanism of how OSA influences the cardiac function and clinical outcomes after STEMI remains uncertain. Previous studies have pointed out that OSA accompanied with sleep fragmentation, nocturnal hypoxemia, and sympathetic activation may be closely related to cardiac rhythm disturbances and ST-segment changes [42]. OSA was also reported to be associated with increased CRP levels, coagulation abnormalities, and activated platelet function [43]. Moreover, OSA was thought to be an important factor in the systemic inflammation, oxidative stress, and endothelial dysfunction [44]. All these results demonstrated that OSA may influence the clinical outcomes and cardiac function in STEMI patients through long-term adverse hemodynamic changes.

There are several limitations in our study. The relatively small sample size limited the evaluation of the association of OSA with cardiac function and clinical outcomes in clinical practice. Therefore, additional prospective data are needed in a larger study population to confirm our findings. Patients in our study were divided according to presence (AHI ≥ 5) or absence (AHI < 5) of OSA, and the stratification should be more refined according to the severity of OSA. Moreover, we did not investigate the exact underlying pathophysiological mechanism, and data on OSA-related symptoms were not obtained since the associated symptoms may be important in the recovery of cardiac function and clinical outcomes. Further studies should be focused on the molecular mechanisms of how OSA regulates the cardiac function and clinical outcomes in STEMI patients. Despite the limitations of our approach, the results of this study provided effective proof that the OSA is independent of other known risk factors in the clinical outcomes and closely related to the cardiac function in STEMI patients.

## 5. Conclusions

In summary, 44.9% of the patients admitted with STEMI have OSA. OSA is a powerful predictor of a lower event-free survival rate and carries a negative prognostic impact on cardiac function in STEMI patients. The underlying pathophysiological mechanism needs to be explored further.

## Figures and Tables

**Table 1:** Baseline characteristics of enrolled patients.

Characteristic	OSA group (*n* = 89)		Non-OSA group (*n* = 109)		*P* value
	Number	(%)	Number	(%)	
Age (mean ± SD)	61.45 ± 7.30		61.57 ± 7.25		0.912
Gender					0.362
Male	61	(68.5)	71	(65.1)	
Female	28	(31.5)	38	(34.9)	
Hypertension					0.122
Yes	54	(60.7)	56	(51.4)	
No	35	(39.3)	53	(48.6)	
Diabetes mellitus					0.413
Yes	25	(28.1)	28	(25.7)	
No	64	(71.9)	81	(74.3)	
Lipid disorders					0.052
Yes	15	(16.9)	9	(8.3)	
No	74	(83.1)	100	(91.7)	
CKD					0.495
Yes	8	(9.0)	11	(10.1)	
No	81	(91.0)	98	(89.9)	
Tobacco use					0.378
Yes	23	(25.8)	25	(22.9)	
No	66	(74.2)	84	(77.1)	
Drinking habit					0.480
Yes	15	(16.9)	17	(15.6)	
No	74	(83.1)	92	(84.4)	
Prior PCI					0.698
Yes	1	(1.1)	1	(0.9)	
No	88	(98.9)	108	(99.1)	
Prior CABG					0.039
Yes	4	(4.5)	0	(0)	
No	85	(95.5)	109	(100)	
cTnT	4.58 ± 3.66		4.72 ± 3.82		0.794
DAPT adherence					0.173
Yes	76	(85.4)	99	(90.8)	
No	13	(14.6)	10	(9.2)	
Socioeconomic status					0.048
Low	9		18		
Medium	68		50		
High	32		21		
Abdominal circumference (mean ± SD)	90.0 ± 9.4		87.4 ± 8.9		0.046
BMI (mean ± SD)	28.8 ± 4.5		24.6 ± 3.1		< 0.001
Aspirin					0.334
Yes	81	(91.0)	96	(88.1)	
No	8	(9.0)	13	(11.9)	
Clopidogrel					0.283
Yes	83	(93.3)	98	(89.9)	
No	6	(6.7)	11	(10.1)	
ACEI/ARB					0.523
Yes	46	(51.7)	57	(52.3)	
No	43	(48.3)	52	(47.7)	
*β*-blockers					0.478
Yes	51	(57.3)	64	(58.7)	
No	38	(42.7)	45	(41.3)	
Statins					0.378
Yes	79	(88.8)	94	(86.2)	
No	10	(11.2)	15	(13.8)	
Nitrates					0.069
Yes	36	(40.4)	32	(29.4)	
No	53	(59.6)	77	(70.6)	
CCB					0.589
Yes	9	(10.9)	11	(10.1)	
No	80	(88.1)	98	(89.9)	
Diuretics					0.159
Yes	6	(6.7)	3	(2.8)	
No	83	(93.3)	106	(97.2)	
Digitalis					0.375
Yes	5	(5.6)	4	(3.7)	
No	84	(94.4)	105	(96.3)	

CKD: chronic kidney disease; PCI: percutaneous coronary intervention; CABG: coronary artery bypass grafting; cTnT: cardiac troponin T; DAPT: dual antiplatelet therapy; BMI: body mass index; ACEI: angiotesin converting enzyme inhibitors; ARB: angiotensin II receptor blockers; CCB: calcium channel blockers.

**Table 2:** Comparison of coronary angiography results of enrolled patients.

	OSA group (*n* = 89)	Non-OSA group (*n* = 109)	*P* value
Coronary lesions			
Single-vessel disease (*n*)	21	30	0.322
2-vessel disease (*n*)	16	32	0.044
3-vessel disease (*n*)	51	47	0.033
Degree of coronary stenosis			
90% < *D* < 100% (*n*)	54	52	0.047
70% < *D* ≤ 90% (*n*)	20	24	0.537
50% ≤ *D* ≤ 70% (*n*)	15	33	0.021
Type of coronary vasculature			
Dominant RCA (*n*)	77	90	0.288
Classic (*n*)	8	13	0.334
Dominant LCA (*n*)	4	6	0.505
Residual stenosis			
Nonreperfusion (*n*)	4	6	0.505
≤20% (*n*)	15	19	0.534
>20% (*n*)	62	84	0.155
Preprocedural TIMI flow			
Nonreperfusion (*n*)	4	6	0.505
0 (*n*)	46	62	0.279
1 (*n*)	22	17	0.077
2 (*n*)	11	14	0.547
3 (*n*)	6	10	0.361
Postprocedural TIMI flow			
Nonreperfusion (*n*)	4	6	0.505
0 (*n*)	6	6	0.471
1 (*n*)	7	5	0.253
2 (*n*)	14	16	0.496
3 (*n*)	58	76	0.298
Revascularization			
PTCA (*n*)	20	17	0.147
PTCA + Stent (*n*)	65	89	0.101
CAG only (*n*)	4	6	0.505
Number of stents (*n*)	67	108	N.S.
Stent diameter (mm)	3.08 ± 0.43	3.09 ± 0.49	0.872
Stent length (mm)	28.5 ± 13.4	28.5 ± 13.6	0.977
Lesion length (mm)	29.3 ± 13.7	29.2 ± 13.5	0.943

LCA: left coronary artery; RCA: right coronary artery; *D*: diameter; N.S.: not suited; CAG: coronary angiography; PTCA: percutaneous transluminal coronary angioplasty; TIMI: Thrombolysis in Myocardial Infarction Trial.

**Table 3:** Cardiac structure and function in the study population.

	OSA group (*n* = 89)	Non-OSA group (*n* = 109)	*P* value
Cardiac structure			
LVM (g)	144 ± 25	133 ± 34	0.012
IVS (cm)	0.91 ± 0.17	0.85 ± 0.14	0.015
PWT (cm)	0.85 ± 0.11	0.82 ± 0.13	0.045
LAD (cm)	3.45 ± 5.35	3.53 ± 4.98	0.268
LVDd (cm)	4.78 ± 0.45	4.77 ± 0.49	0.857
LVDs (cm)	2.95 ± 4.90	2.94 ± 4.98	0.966
WMA (%, *n*)	67.4 (60)	64.2 (70)	0.375
Cardiac function			
LVEF (%)	60.7 ± 13.4	64.4 ± 11.4	0.038
Peak *E* (cm/s)	74.2 ± 17.3	72.1 ± 26.5	0.516
Peak *A* (cm/s)	75.4 ± 15.0	64.6 ± 21.4	<0.001
DT (s)	2.04 ± 0.18	1.97 ± 0.19	0.016
IVRT (s)	0.10 ± 0.07	0.09 ± 0.08	0.037
*s*′ (cm/s)	7.27 ± 1.26	7.25 ± 1.25	0.930
*e*′ (cm/s)	7.00 ± 1.53	7.60 ± 2.44	0.047
*a*′ (cm/s)	6.99 ± 0.16	6.78 ± 0.17	<0.001
*E*/*A* ratio	1.02 ± 0.32	1.22 ± 0.55	0.004
*e*′/*a*′ ratio	1.00 ± 0.22	1.12 ± 0.36	0.006
*E*/*e*′ ratio	10.6 ± 1.11	9.32 ± 1.83	<0.001

LVM: left ventricular mass; IVS: thickness of interventricular septum; PWT: thickness of posterior wall of left ventricle; LAD: left atrial dimension; LVDd: left ventricular end-diastolic dimension; LVDs: left ventricular end-systolic dimension; WMA: wall motion abnormality; LVEF: left ventricular ejection fraction; Peak *E*: the peak of early diastolic velocities; Peak *A*: the peak of late diastolic velocities; DT: the deceleration time of the peak *E* velocity; IVRT: isovolumic relaxation time; *s*′: systolic motion wave velocities; *e*′: early diastolic mitral annulus velocities; *a*′: late diastolic mitral annulus velocities.

**Table 4:** Results of multivariate Cox regression analysis with MACE and cardiac death.

Characteristics	MACE		Cardiac death	
	Hazard ratio (95% CI)	*P* value	Hazard ratio (95% CI)	*P* value
Gender	1.124 (0.538–2.351)	0.756	1.125 (0.283–4.477)	0.868
Old age (≥65 years)	0.871 (0.409–1.853)	0.720	1.382 (0.394–4.846)	0.613
Hypertension	1.310 (0.605–2.837)	0.493	0.675 (0.184–2.477)	0.553
Diabetes mellitus	3.088 (1.495–6.378)	0.002	9.057 (2.079–39.454)	0.003
Lipid disorders	2.279 (0.932–5.572)	0.071	1.395 (0.127–15.373)	0.786
CKD	1.010 (0.267–3.818)	0.988	0.252 (0.015–4.303)	0.341
Tobacco use	1.421 (0.526–3.839)	0.488	1.630 (0.403–6.596)	0.493
Drinking habit	0.779 (0.235–2.582)	0.682	0.253 (0.036–1.776)	0.167
OSA	3.946 (1.631–9.544)	0.002	11.189 (1.562–80.177)	0.016
Prior PCI	6.036 (0.636–57.273)	0.117	0.100 (1.130–1.150)	0.995
Prior CABG	1.970 (0.372–10.437)	0.425	0.100 (1.132–1.187)	0.986
LVEF	0.433 (0.962–11.017)	0.853	0.967 (0.929–1.007)	0.105
*E*/*e*′ ratio	0.865 (0.719–1.041)	0.125	0.966 (0.675–1.381)	0.848
cTnT	0.999 (0.907–1.102)	0.991	1.189 (0.971–1.457)	0.094
WMA	1.231 (0.523–2.898)	0.634	2.294 (0.355–14.832)	0.383
DAPT adherence	0.126 (0.048–0.333)	<0.001	0.025 (0.004–0.160)	<0.001
Abdominal circumference	1.015 (0.974–1.058)	0.471	0.987 (0.914–1.066)	0.745
Socioeconomic status	1.647 (0.931–2.913)	0.086	1.118 (0.389–3.211)	0.835
BMI	0.940 (0.848–1.042)	0.239	0.933 (0.744–1.170)	0.547

CKD: chronic kidney disease; OSA: obstructive sleep apnea; PCI: percutaneous coronary intervention; CABG: coronary artery bypass grafting; LVEF: left ventricular ejection fraction; cTnT: cardiac troponin T; WMA: wall motion abnormality; DAPT: dual antiplatelet therapy; BMI: body mass index.
